# Post-Vaccination Assessment of Peste Des Petits Ruminants in Sheep and Goats in the United Arab Emirates

**DOI:** 10.3390/vetsci12100991

**Published:** 2025-10-14

**Authors:** Yassir M. Eltahir, Mervat Mari. Al Nuaimat, Oum Keltoum Bensalah, Ebrahim Osman, Diya S. Al-Ramamneh, Rashid A. Khan, Naema A. Alsuwaidi, Meera Saeed. Mohamed, Kaltham Kayaf, Sameera Ismaeil, Fatmah Yaaqeib, Mahmoud Abdelfatah, Ahmed Tharwat, Mohamed Antar, Mohammed Abd Elmottalib. Kheir, Assem S. Abdelazim, Rafeek Koliyan, Mohamed Moustafa. Abdelhalim

**Affiliations:** 1 Animals Extension and Health Services Division, Abu Dhabi Agriculture and Food Safety Authority (ADAFSA), Abu Dhabi 52150, United Arab Emirates; 2 Animal Development and Health Department, Ministry of Climate Change and Environment, Dubai P.O. Box 1509, United Arab Emirates; 3 Animal Wealth Development Division, Abu Dhabi Agriculture and Food Safety Authority (ADAFSA), Abu Dhabi 52150, United Arab Emirates; 4 Biosecurity Affairs Division, Development and Innovation Sector, Abu Dhabi Agriculture and Food Safety Authority (ADAFSA), Abu Dhabi P.O. Box 52150, United Arab Emirates; 5 National laboratories Department, Ministry of Climate Change and Environment, Dubai P.O. Box 1509, United Arab Emirates; 6 Eastern Region Department, Ministry of Climate Change and Environment, Dubai P.O. Box 1509, United Arab Emirates; 7 Middle Region Department, Ministry of Climate Change and Environment, Dubai P.O. Box 1509, United Arab Emirates; 8 Northern Region Department, Ministry of Climate Change and Environment, Dubai P.O. Box 1509, United Arab Emirates; 9 Dubai Region Department, Ministry of Climate Change and Environment, Dubai P.O. Box 1509, United Arab Emirates

**Keywords:** eradication, PPR, mass vaccination, seroprevalence, UAE, vaccine efficacy

## Abstract

**Simple Summary:**

Peste des petits ruminants (PPR) post-vaccination monitoring was carried out at a national level in the United Arab Emirates (UAE) in 2024 in accordance with the Global Control and Eradication Strategy (GCES) guidelines. Two serosurveys conducted pre and post the national mass PPR vaccination revealed the use of an efficient PPR vaccine to immunize sheep and goats which resulted in higher post-vaccination herd immunity than the threshold recommended by the PPR GCES. Mass vaccination of sheep and goats with a target over 95% coverage is recommended for the next three years to achieve the sustained desired level of immunity at the holding level.

**Abstract:**

Background: Peste des petits ruminants (PPR) is an acute or subacute contagious trans-boundary viral disease causing high morbidity and mortality in domestic and wild small ruminants. The national UAE-PPR control and eradication plan follows the PPR Global Control and Eradication Strategy (PPR GCES) and relies on the annual mass vaccination of small ruminants to eradicate the disease from the country by 2030. Despite the immunization effort against PPR, the vaccination coverage reached 65% at maximum, which necessitates conducting a post-vaccination evaluation (PVE) study at the national level. Methods: Using multistage random sampling to assess the PPR vaccine and vaccination effectiveness, protocol (2) of the PPR GCES, using two serosurveys; serosurvey (1) (pre-vaccination) at day 0 before vaccination, to assess the primary PPR serological investigation, and serosurvey (2) at (30–90) days post-PPR vaccination, to evaluate the immune response, were carried out from September to December 2024 across the seven Emirates of the UAE. The nucleoprotein-based competitive enzyme-linked immunosorbent assay (c-ELISA) was used to detect PPR antibodies in a total of 1592 and 1589 sera samples collected, respectively, before and after vaccination from different (*n* = 163) sheep and goats holdings (epi-unit) distributed in the different Emirates of the UAE. Results: In serosurvey (1). prior to vaccination, out of the total 1592 samples tested (839 goats and 753 sheep), 833 animals (52.32%) were found to be seropositive for PPR antibodies. In contrast, in serosurvey (2), after vaccination, 1490 (93.77%) animals were found to be seropositive out of the total 1589 small ruminants (825 goats and 764 sheep) tested by c-ELISA. A statistically significant increase (41.45%) in the overall seroprevalence from (52.32%) pre-vaccination to (93.77%) post-vaccination was observed. Post-vaccination, 93.87% (*n* = 153) of the vaccinated epi-units achieved more than 70% seroprevalence compared to 43.56% (*n* = 71) before vaccination. Prediction analysis showed that all the seven UAE Emirates require 1.2 years maximum to reach 100% immune-protection levels. Conclusions: An efficient PPR vaccine was used to immunize small ruminants in the UAE. Higher (89.47–100%) post-vaccination herd immunity than the threshold recommended by the PPR GCES (>80% immunity) was attained, which can efficiently break the spread of PPRV within the UAE. To enhance the eradication of PPR I the UAE, conducting mass vaccination campaigns targeting over the (95%) immunization coverage of eligible animals for the next three years is recommended to attain the requested sustained (>80%) immunity at the animals holding level.

## 1. Introduction

Peste des petits ruminants (PPR) described first in West Africa in 1942 is an acute or subacute contagious trans-boundary viral disease affecting domestic and wild small ruminants. It is characterized by anorexia, fever, necrotic stomatitis, diarrhea, ocular and nasal purulent discharge, and respiratory distress, with morbidity and mortality rates that can reach up to 100% and 76%, respectively. PPR is caused by the PPR virus (PPRV), an enveloped virus with a single-stranded, negative-sense RNA genome that belongs to the Paramyxoviridae family and the Morbillivirus genus [1,2]. Globally, PPR is endemic in many regions of Africa, the Middle East and Asian countries and has recently been reported in some European countries including Romania, Greece, and Hungary resulting in the suspension of their free status [3,4]. The disease is economically significant, causing the death of small ruminants and affecting the lives of livestock keepers in low- and middle-income countries. It is also causing devastating outbreaks in susceptible wildlife, threatening biodiversity. Therefore, the Food and Agriculture Organization (FAO) and the World Organization for Animal Health (WOAH) launched the PPR Global Control and Eradication Strategy (PPR GCES) in 2015 in order to completely eliminate PPR from the globe by the year 2030 under the Global Eradication Program (GEP) [5].

The PPR GCES relies on a four-stage (1–4) stepwise approach, each step corresponding to a combination of decreasing levels of epidemiological risk and increasing levels of prevention and control efforts; these include assessment, control, eradication, and post eradication stages. In stage 1, assessing the local epidemiological situation in a country is required; stage 2 is dedicated to controlling PPR infection, through vaccination campaigns informed by surveillance; in stage 3, the eradication of PPR should be enhanced by strengthening surveillance and preventive measures; and lastly, in stage 4, vaccination must be ceased, with evidence proving that PPRV is no longer circulating either at zonal or national levels [6].

In the United Arab Emirates (UAE), PPR is endemic where PPRV lineage III was first reported in wildlife in 1986 [7]. Later, in 200, the Asian lineage IV was also detected in wildlife, while the first characterization of lineage IV in goats was recently published in 2023 [8,9]. The total livestock population in the UAE is around 5 million, comprising 4.13 million sheep and goats which represent 86% of the total livestock in the country, 550,000 camels, and 110,000 cattle. PPR is a notable disease in the UAE, and since its first official report to the World Animal Health Information System (WAHIS) in 2005, PPR has been reported regularly in the UAE to affect domesticated animals and wildlife [10]. To date, a total number of 81 PPR outbreaks were reported to WAHIS [11]. The national UAE-PPR control and eradication plan adopted in 2016 follows the PPR GCES and relies on the mass vaccination of small ruminants strategy. Annual vaccination campaigns to vaccinate sheep, goats, and captive antelopes, with the target of 80% vaccination coverage, are conducted using the live PPR vaccine strain (Nig 75/1) supplied by MEVAC, (El Salhya El Gdeda, Egypt). All animals older than 3 months are vaccinated, and are then revaccinated every three years [9]. To maximize the efficiency of the vaccination campaigns, the Abu Dhabi Agriculture and Food Safety Authority (ADAFSA) has developed a risk-based approach for the mobilization of the PPR vaccination teams in the field, relying on artificial intelligence. This has allowed vaccinators to reach a significant number of flocks which were not vaccinated by previous traditional vaccination campaigns [12].

Despite the immunization effort of small ruminants against PPR since 2016 in the UAE, the vaccination coverage across the country reached 65% at maximum. Post-vaccination evaluation (PVE) is partially implemented in the UAE but without covering the whole country, and neither active surveillance nor systematic seromonitoring to assess the effectiveness of the PPR vaccination program at a national level was conducted previously. Therefore, this cross-sectional serological survey was carried out in 2024 to determine PPR pre- and post-vaccination antibody status in sheep and goats at a national level. This, in turn, will fulfill some requirements of the national UAE-PPR control and eradication plan, improve the UAE stage in the PPR GCES, and enhance the efforts towards eradicating the disease from the country by 2030.

## 2. Materials and Methods

### 2.1. Study Area

**Figure 1:**
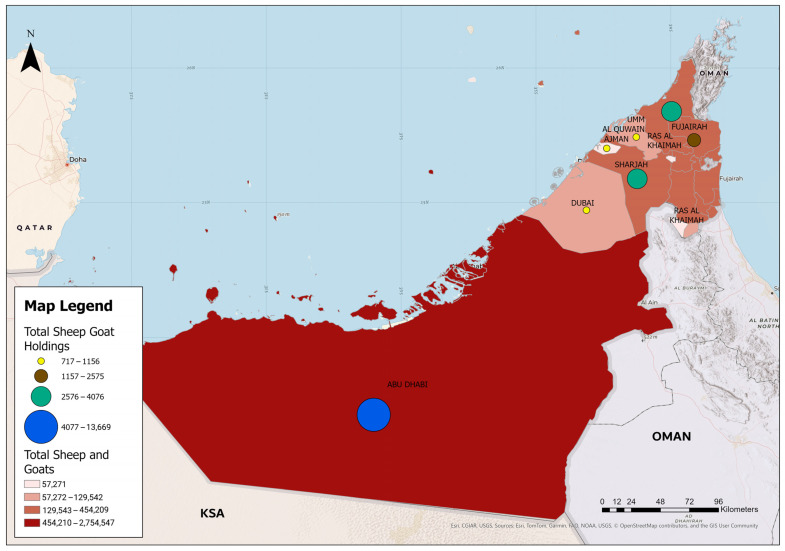
Density of the small ruminant (Sheep and Goats) populations in the different Emirates of the UAE.

The PPR-PVE in sheep and goats in the UAE was carried out during September–December 2024 at a national level throughout the seven Emirates of the country. These included Abu Dhabi, Dubai, Sharjah, Ajman, Umm Al Quwain, Ras Al Khaimah, and Fujairah Emirates. The total number of sheep and goats in the UAE is 4,133,551 animals, located in 26,689 different holdings, which are distributed among the Emirates with the corresponding (66.64%, 3.13%, 10.99%, 1.39%, 2.54%, 8.64% and 6.68%) percentages, respectively (Table 1, Figure 1). The small ruminants farming system in the UAE is stationary animal husbandry where animals remain in holding throughout the year. Animals are moved only for slaughter, to the animal markets, and for exportation or re-exportation purposes. The climate of the UAE is arid, with very high summer (June–September) temperatures and humidity reaching 46 °C and 100%, respectively. In winter (December–March), over 80% of the annual rain occurs and temperature ranges between 14 °C and 23 °C [13].

### 2.2. Samples Collection and Vaccination

The PPR-PVE tool is a companion to the PPR GCES. The tool includes three different protocols (1–3), each with specific PVE objectives. In this study, protocol (2) was followed to assess the immune response to PPR vaccination as a proxy for the effectiveness of the vaccination campaign. It comprises the implementation of two serosurveys, which were as follows: serosurvey (1) at day (0) before vaccination, for assessing the primary PPR serological investigation, and serosurvey (2) at (30–90) days after vaccination for the post-vaccination immune response assessment. Accordingly, using multistage random sampling with a 95% confidence level, a precision level of 5% and an expected 50% seroconversion, as there was no previous estimate, sample sizes were adjusted in the study population. All animals sampled were above 6 months of age and a maximum number of (8–10) animals (equally divided among sheep and goats whenever applicable) samples were collected from each epi-unit [5]. For the serosurvey (1), blood from 1592 small ruminants, including 839 goats and 753 sheep, were collected from randomly selected (*n* = 163) small ruminants holdings (epi-units) that included (76, 9, 29, 4, 7, 21 and 17) holdings in Abu Dhabi, Dubai, Sharjah, Ajman, Umm Al Quwain, Ras Al Khaimah and Fujairah Emirates, respectively (Table 2). After the completion of the samples collection for serosurvey (1), a joint national mass vaccination campaign against PPR, with a target of 80% coverage, was carried out in the seven Emirates using the PPR strain Nigeria 75/1 in September–October 2025. Vaccination in the Emirate of Abu Dhabi was carried out by Abu Dhabi Agriculture and Food Safety Authority (ADAFSA) Animals Extension and Health Services division, while vaccinations in the remaining Emirates were deployed by the Ministry of Climate Change and Environment (MOCCAE) veterinary sections or the local municipalities veterinary sections. During the vaccination campaign, the cold chain was maintained from the store to the field by keeping the vaccine at (2–8 °C) and using it within one hour of reconstitution. After a total of 30-days post PPR vaccination, a total number of 1589 blood samples (829 and 768) from goats and sheep, respectively, were then randomly collected from (*n* = 163) vaccinated epi-units (Table 3). The collected blood samples were labeled, serum was separated from blood by centrifuging at 5000 rpm for 5 min for each sample, and the separated sera were transported in an ice-cool shipment box to ADAFSA veterinary laboratory and were stored at −20 °C until further use.

### 2.3. Serology

The Competitive ELISA kit (ID Screen^®^PPR Competition, Innovative Diagnostics, Montpellier, France) for the detection of anti-PPRV nucleoprotein antibodies in sheep and goats was used in the serosurveys, according to manufacturer’s instructions. Results were read at a wavelength of 450 nm. Test results were considered reliable if the average value of the ODNC > 0.7 and the ratio of the average values of the ODPC/ODNC < 0.3. The S/N percentage (S/N%) value was calculated for each sample using the formula sample ODsample/ODNC × 100%. Samples showing a ratio of S/N% ≤ 50% were considered positive, whereas S/N% > 60% were considered negative. Samples with a ratio of 50% < S/N% ≤ 60% were considered doubtful and were not included in the final results [14].

### 2.4. Vaccine Efficacy and Vaccination Effectiveness

Vaccine efficacy was calculated in terms of the percentage of seroconversion thirty days post receiving a single dose of the PPR vaccination, while vaccine effectiveness was obtained by multiplying the percentage of vaccination coverage [(number of animals vaccinated/total eligible animals for vaccination) × 100] in the study population by the percentage efficacy of the vaccine as previously described [15].

### 2.5. Annual Growth Rate of Immuno-Protection Threshold

Using a linear model based on the observed annual increase in seroprevalence from pre-vaccination to post-vaccination levels, we estimated the number of years required for each Emirate to achieve immune-protection thresholds of 70%, 80%, 90%, and 100%. The predictive calculations were performed using the formula:

Years to Target=(T−a)/GR

where T = the target seroprevalence (%), a = the initial (pre-vaccination) seroprevalence (%), GR = the annual increase in seroprevalence (%) = (b − a)/N, b = post-vaccination seroprevalence (%), and N = number of years between surveys (typically N = 1) [16].

### 2.6. Statistical Analysis

The seroprevalence of PPR in the UAE was calculated in serosurvey (1) and (2) studies, based on the number of positive animals versus the number of tested animals. Simple descriptive statistics for the frequencies and Pearson’s chi-squared test were used to understand the association between the presence of PPRV antibodies in sheep and goats across the Emirates as well as between the sheep and goats within each Emirate. Differences were considered significant and highly significant when *p*-values were less than 0.05 and 0.01, respectively, using the chi-squared test [17].

## 3. Results

### 3.1. PPR Antibody Seroprevalence Before Vaccination

In the initial serosurvey (1), before vaccination, out of the total 1592 samples tested (839 goats and 753 sheep) that originated from (*n* = 163) epi-units distributed in the different seven Emirates of the UAE, 833 animals (52.32%) were found to be seropositive for PPR antibodies by c-ELISA. Specifically, goats showed (54.95%) seroprevalence, while a 49.40% seroprevalence was observed in sheep. The total PPR seroprevalences varied significantly (chi-squared test: *p* < 0.001) across the different Emirates. The lowest (41.40%) seroprevalence was detected in 76 animal holdings tested in Abu Dhabi Emirate, whereas the highest (77.07%) seroprevalence in 21 holdings was observed in Ras Al-Khaimah Emirate. The lowest seroprevalence in goats (40%) was observed in Umm Al-Quwain Emirate, whereas the highest (80.95%) seroprevalence in the same animal species was observed in Ras Al-Khaimah Emirate. In sheep, the lowest seroprevalence (35.60%) was observed in Abu Dubi Emirate, whereas the highest (73%) seroprevalence in sheep was observed in Ras Al-Khaimah Emirate (Table 2). Logistic regression analysis confirmed that both Emirate and species (*p* < 0.001) were significant predictors of seropositivity prior to vaccination. Correlation analysis revealed a moderate positive association between seroprevalence and the number of holdings sampled (Pearson’s r = 0.61, *p* < 0.01).

### 3.2. PPR Post-Vaccination Seroprevalence

Out of the total 1589 small ruminants (825 goats and 764 sheep) tested by c-ELISA in serosurvey (2), after PPR mass vaccination, 1490 (93.77%) animals were found to be seropositive for PPR antibodies. In total, goats and sheep exhibited 92.61% and 95.03% seroprevalence, respectively, post PPR vaccination. Chi-squared analysis showed a statistically significant increase in seroprevalence in the total (*n* = 163) sheep and goats holdings tested after vaccination across all Emirates (*p* < 0.001). Abu Dhabi Emirate remained the lowest (89.47%), while Dubai (96.67%), Fujairah (97.65%), Umm Al-Quwain (100%), Ajman (95.00%), and Ras Al-Khaimah (96.00%) Emirates all demonstrated high post-vaccination seroprevalence. When seroprevalence was compared among the animal species and Emirates, post-vaccination seroprevalence was highest in Umm Al-Quwain Emirate, reaching 100% for both goats and sheep. The lowest rates were found in Abu Dhabi Emirate, at 88.10% for goats and 91.07% for sheep. Logistic regression confirmed the significant effects of both Emirate (*p* < 0.001) and species (*p* < 0.001) on post-vaccination seropositivity (Table 3).

### 3.3. Assessing Effectiveness of Vaccination Campaign

**Figure 2:**
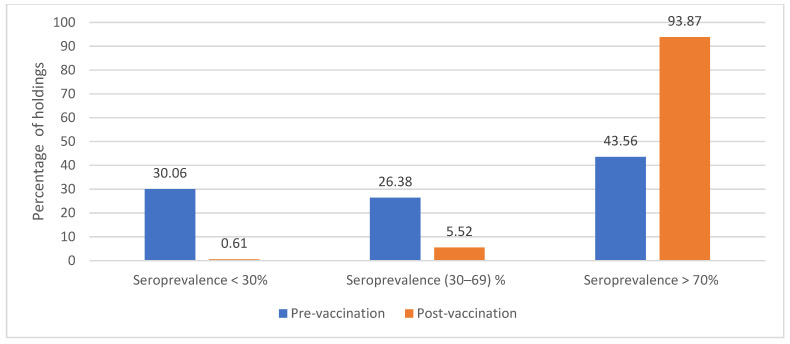
Percentages of distribution of PPR antibodies seroprevalence in animal holdings (*n* = 163) before and after vaccination.

According to the GCES guidelines, if a vaccine is effective at least 50% of the vaccinated epi-units should have a minimum of 70% seropositive animals, whereas vaccination failure is defined when 30% or more of animals within the epi-unit are seronegative [5]. Thus, when evaluating the PPR vaccination campaign effectiveness, it was revealed that 30.06% and 26.38% (*n* = 49 and 43) epi-units had less than 30% and (30–69%) seroprevalences, respectively, before vaccination. In contrast, after vaccination, a single holding (0.61%) had a seroprevalence below 30%, while 93.87% (*n* = 153) of the vaccinated epi-units achieved more than 70% immune response, compared to 43.56% (*n* = 71) before vaccination (Figure 2). Marked (41.45%) overall increase in seroprevalence from (52.32%) before vaccination to (93.77%) after vaccination was observed in the total epi-units (*n* = 163) in the study, representing statistically significant improvement (chi-squared test: *p* < 0.001).

### 3.4. Estimation of Time to Reach Immuno-Protection Threshold

When the number of years required for each Emirate to achieve immune-protection thresholds of 70%, 80%, 90%, and 100% were predicted, it was found that Emirates such as Ajman and Ras Al-Khaimah had already surpassed the 70% benchmark prior to the campaign, requiring no additional time to reach that level. For the remaining Emirates—Abu Dhabi, Dubai, Sharjah, Umm Al-Quwain, and Fujairah—the model predicted that all four protection thresholds would be achieved within 1.2 years or less. At the nationwide level, it was revealed that the UAE needs (0.4, 0.7, 0.9 and 1.2) years to reach 70%, 80%, 90%, and 100% immunity against PPR, respectively (Table 4).

## 4. Discussion

The wide geographical endemic occurrence of PPR across the Gulf Cooperation Council (GCC) countries, the Middle East, and major zones of sub-Saharan Africa and Asia, coupled with its negative socio-economic impact, has prompted progressive control and eradication efforts at both regional and global levels [17,18]. Accordingly, in March 2015, WOAH and FAO officially adopted the PPR GCES. The first five-year phase of the PPR-GEP was launched in 2017, whereas the second and third phases were launched in November 2022 as the “Blueprint for PPR eradication from the globe by 2030” [5,19].

Like rinderpest, PPR exhibits several eradicable disease characteristics. These include the following: PPR is caused by a single viral serotype for which the current PPR vaccines can induce protection against all viral lineages and for which immunity is lifelong following natural infection or vaccination [6]; the primarily means of transmission of the disease is via direct contact [20]; the virus is fragile outside the host and sensitive to heat and sunlight [21]; infected animals are infectious for a short period and there is no carrier state [22,23]; and there is no established evidence of virus maintenance in wildlife or subsequent transmission to domestic small ruminants [24].

The PPR GCES encourages countries to participate in the regional PPR roadmaps, which are designated based on FAO and WOAH regional divisions and epidemiological considerations. In this context, nine different regions were established using an epi-zone approach, which categorizes together countries with similar PPR epidemiological situations to enhance the cooperation and coordination of PPR eradication efforts. Each region roadmap is targeted by periodical meetings and overseen by a Regional Advisory Group (RAG). The Middle Eastern Region Roadmap encompasses fifteen countries, namely Bahrain, Iran, Iraq, Jordan, Kuwait, Lebanon, Sultanate of Oman, Palestine, Qatar, Syria, Saudi Arabia, the United Arab Emirates (UAE), Egypt, Yemen, and Israel [25,26,27].

Currently, the UAE is in stage 3 of the stepwise approach outlined in the PPR GCES. Due to lifelong immunity following vaccination, the PPR GCES is largely dependent on the strategic mass vaccination of small ruminant populations. Therefore, the UAE’s PPR control and eradication plan, adopted in 2016, also emphasizes the mass vaccination of small ruminants, early laboratory detection, passive and active surveillance, and post-vaccination assessment as the main principles of its success to eradicate the disease from the country by 2030 [9].

Passive PPR surveillance in the UAE is conducted through clinical monitoring and a rapid alert and response system for both domestic and wild animals as part of the National Biosecurity Notification System. In contrast, active PPR surveillance is planned when the vaccination is ceased at the future stages of the eradication process. Since 2016, annual mass vaccination campaigns targeting sheep, goats over three months of age, and captive antelopes, with a target coverage of 80% vaccination, are conducted from September to December to coincide with the lambing seasons using the live PPR vaccine strain (Nig 75/1). From 2019 to 2022, ADAFSA has developed a risk-based approach for the mobilization of PPR vaccination teams relying on artificial intelligence in Abu Dhabi Emirate, which harbor 66.64% of the total small ruminant population in UAE. This approach enables the selective vaccination of animal holdings based on the five estimated risk factors, (i) time since last vaccination, (ii) forecasted number of untagged animals, (iii) current flock size, (iv) proximity (<5 km) to recent outbreaks, and (v) predicted vaccine rejection or non-compliance. Implementation of this approach enhanced vaccination efficiency, increased flock coverage, shortened campaign durations, and reduce strain on human and logistic resources [12].

In stages 2 and 3 of the PPR GCES, vaccination is one of the principal measures of controlling PPR. The purpose of the PPR vaccination program is to support disease control (stage 2) or eradication (stage 3) of the disease. In both stages, the (i) vaccine attributes, (ii) vaccine delivery and coverage, and (iii) assessment of the immune response, need to be regularly monitored by conducting PVE. Therefore, this study aimed to evaluate the immune response to PPR vaccination as a proxy for the effectiveness of the mass vaccination campaign in the UAE, thereby providing crucial insights into population-level immunity, vaccine efficacy, and effectiveness assessment at the field level, with the result of refining the national UAE-PPR control and eradication plan to achieve its PPR-free status target [28].

In this study, following protocol (2) of the PPR-PVE included in the PPR GCES, two serosurveys were conducted at a national level using multistage random sampling. These included the following: serosurvey (1), pre-vaccination, for assessing the primary PPR serological investigation, and serosurvey (2), one-month post-vaccination, for the PVE. Prior to the PPR mass vaccination campaign in the UAE, the overall PPR antibodies seroprevalence in sheep and goats above 6 months of age in the targeted epi-units (*n* = 163) distributed among the different seven Emirates of the UAE was (52.32%). This result is in agreement with seroprevalence reported pre-vaccination in the same animal age group in India [16]. In the UAE, previous PPR seroprevalence status due to either infection or vaccination is not reported. Thus, the results obtained here represent the first pre-vaccination baseline seroprevalence of PPR in the country. The potential exposure of the tested animals to the virus cannot be ruled out as the vaccine or the test used did not differentiate between the infected and vaccinated animals. However, the observed marked decrease in PPR outbreaks up to one outbreak per annum since the implementation of the UAE-PPR control and eradication plan in 2016 [11] and the clinical monitoring of outbreaks during pre- and post-vaccination serum sampling without the detection of clinical infection or laboratory-confirmed cases suggest that the observed pre-vaccination population immunity was due to the effort of the previous annual mass or risk-based vaccination campaigns in the UAE, rather than to natural infection.

The seroprevalences of PPR before vaccination varied significantly (*p* < 0.05) across the different Emirates where seroprevalences below 70% (41.40%, 46.67%, 50%, 55.17% and 64.12%) were detected in Abu Dhabi, Dubai, Umm Al–Quwain, Sharjah, and Fujairah Emirates, respectively, while high seroprevalences (70% and 77.07%) were observed in Ajman and Ras Al-Khaimah Emirates, respectively. The low seroprevalence (<70%) detected in animals holdings located in five out of seven Emirates under the study indicates the weaning of herd immunity to a lower level than that recommended by the PPR GCES, which entails 80% herd immunity for an efficient protection barrier against the disease. Furthermore, the observed prior vaccination seroprevalence (<30%) in 30.06% (*n* = 49) of the epi-units in the study in different Emirates could be due to the dynamic population of small ruminants as well as the collected stratified samples, which were from animals after one year of vaccination before the 2024 mass vaccination campaign was conducted [16]. Immunity duration in PPR-vaccinated small ruminants is likely to be 2–3 years long; however, the rapid herd turnover in these animal species leads to the potential for vaccination coverage to decrease by as much as 25% annually, or even faster, if non-vaccinated sheep and goats, including newborns, are brought into the animal unit. Similarly, the withdrawal of vaccinated animals from vaccinated holdings could dilute an already-formed herd immunity [16,28].

The overall (52.32%) observed pre-vaccination seroprevalence ranged from (41.40 to 77.07%) across all Emirate; however, only 62% (*n* = 101) of the epi-units exhibited seroprevalence > 37%, which is considered insufficient to prevent viral circulation in the country. It was observed that in endemic regions, virus transmission could be prevented if 71% of the pastoral village sheep and goat populations were permanently kept with seroprevalence above 37% [29]. These findings underscore the need to expand vaccination coverage to reach a higher target (>95% coverage) of animals and holdings nationwide.

The national PPR-PVE carried out one month after the mass vaccination campaign revealed a significantly (*p* < 0.05) marked increase (41.45%) in the overall seroprevalence from (52.32%) before vaccination to (93.77%) after vaccination, suggesting high vaccination coverage. A total 93.87% (*n* = 153) of the vaccinated epi-units achieved post-vaccination seroprevalence exceeding 70%. It is reported that for a vaccine to be considered effective, at least 50% of vaccinated epi-units should exhibit ≥ 70% seropositive animals [30]. Thus, such high post-vaccination seroprevalence reported here indicates an effective PPR vaccine was used to immunize small ruminants, with the effectiveness of the mass vaccination campaign in terms of good vaccine management practices, adherence to cold chain protocols, and effective vaccination delivery within the UAE [31]. Similar high post-vaccination seroconversion rates have been reported in Pakistan (96%) one month after vaccination [30] and in Bangladesh (100%) in experimental goats [32]. Moreover, the observed high post-vaccination herd immunity, ranging from 89.47% to 100% in sheep and goats throughout the Emirates, was higher than the herd immunity threshold recommended by the PPR GCES to efficiently break the epidemiological cycle of the virus. This reflects that the small ruminant mass vaccination campaign implemented in 2024 has provided the desired protection levels against PPR in the UAE [33]. In contrast to a survey conducted in Ethiopia six months after vaccination, where high seroconversion rate (93.9%) was attained, it was found that only 68% of animals remained in the originally vaccinated flocks. This population turnover reduced the herd immunity to approximately 64%, falling below the threshold required for PPR control under the GCES [34]. It is also reported that PPRV transmission may persist despite very high vaccination coverage (>90%), if vaccination is biased towards more accessible but lower-risk populations highlighting the importance of identifying high-risk populations for vaccination and supporting the adoption of targeted, risk-based vaccination programs [35]. Furthermore, when annual protection was assessed to predict the number of years of vaccination required to achieve 70% protection, using linear projections based on observed seroprevalence increases, it was shown that Emirates like Ajman and Ras Al-Khaimah had already met this benchmark prior to the mass vaccination campaign conducted in 2024. The rest of the other Emirates would achieve 70% and 100% protection thresholds within 0.6 to 1.2 years of vaccination, respectively. As per the global PPR control strategy, a sustained annual PPR mass vaccination in endemic and high-risk areas should achieve a post-vaccination seroprevalence of 70% within three years of implementation [5,26]. Taking into account the overall observed (52.32%) seroprevalence in sheep and goats before vaccination, a national PPR mass vaccination program with a higher target (>95% coverage) of eligible sheep and goats should be implemented in the UAE for three more years, targeting naïve young populations every six months in high-risk areas to achieve sustained high protection levels (>80%) [30]. The study limitations include that all animals tested were confined to holdings with low mobility and lower infection risk, whereas high risk areas such as slaughterhouses or trading market sites were not included. This underscores the need to identify high-risk populations for PPR vaccination and PVE at a certain stage of PPR eradication in the UAE.

## 5. Conclusions

In conclusion, this study represents the first national PPR-PVE in the UAE in accordance with the PPR GCES guidelines. The two conducted serosurveys before and after the national mass PPR vaccination campaign unveil PPR seroprevalence in sheep and goats in the country. A significant marked increase in the overall seroprevalence was achieved after vaccination, suggesting the use of an efficient PPR vaccine to immunize small ruminants in the UAE. Throughout all Emirates, higher post-vaccination herd immunity than the threshold recommended by the PPR GCES was attained, which can efficiently break the spread of PPRV. To enhance the eradication of PPR in the UAE, conducting mass vaccination campaigns targeting the full coverage of eligible small ruminants over three months of age for the next three years is recommended to achieve the desired PPR GCES level of immunity at the holding level. Other PVE studies are also required to monitor the proportion of increase in animal holdings protection. When the desired holding level seroprevalence is attained with minimal or sporadic outbreaks occurrence, efforts can be focused towards high-risk locations such as animal markets and borders to prevent the introduction of PPR.

## Figures and Tables

**Table 1:** Distribution of total small ruminants in the UAE.

Emirate	Total Sheep and Goats	Total Sheep and Goats Holdings	Percentage of Animals from Total Livestock Population
Abu Dhabi	2,754,547	13,669	66.64
Dubai	129,542	717	3.13
Sharjah	454,209	4076	10.99
Ajman	57,271	780	1.39
Umm Al-Quwain	104,766	1156	2.54
Ras Al-Khaimah	357,032	3716	8.64
Fujairah	276,184	2575	6.68
Total	4,133,551	26,689	100

**Table 2:** PPR seroprevalence pre-vaccination.

Emirates	No. of Holdings Sampled	No. of Samples Screened	Total Goats	Total Sheep	No. of ELISA Positive Goats	No. of ELISA Positive Sheep	Totals ELISA Positive	Average PPR Seroprevalence in Goats (%)	Average PPR Seroprevalence in Sheep (%)	Average PPR Seroprevalence in Sheep and Goats (%)
Abu Dhabi	76	727	404	323	186	115	301	46.04	35.60	41.40
Dubai	9	90	45	45	21	21	42	46.67	46.67	46.67
Sharjah	29	290	145	145	80	80	160	55.17	55.17	55.17
Ajman	4	40	20	20	14	14	28	70.00	70.00	70.00
Umm Al-Quwain	7	70	35	35	14	21	35	40.00	60.00	50.00
Ras Al-Khaimah	21	205	105	100	85	73	158	80.95	73.00	77.07
Fujairah	17	170	85	85	61	48	109	71.76	56.47	64.12
Total	163	1592	839	753	461	372	833	54.95	49.40	52.32

**Table 3:** PPR seroprevalence post-vaccination.

Row Labels	No. of Holdings Sampled	No. of Samples Screened	Total Goats	Total Sheep	No. of ELISA Positive Goats	No. of ELISA Positive Sheep	Totals ELISA Positive	Average PPR Seroprevalence in Goats (%)	Average PPR Seroprevalence in Sheep (%)	Average PPR Seroprevalence in Sheep and Goats (%)
Abu Dhabi	76	731	395	336	348	306	654	88.10	91.07	89.47
Dubai	9	90	45	45	42	45	87	93.33	100.00	96.67
Sharjah	29	288	140	148	134	149	283	95.71	100.68	98.26
Ajman	4	40	20	20	19	19	38	95.00	95.00	95.00
Umm Al-Quwain	7	70	35	35	35	35	70	100.00	100.00	100.00
Ras Al-Khaimah	21	200	105	95	102	90	192	97.14	94.74	96.00
Fujairah	17	170	85	85	84	82	166	98.82	96.47	97.65
Total	163	1589	825	764	764	726	1490	92.61	95.03	93.77

**Table 4:** Estimated years to reach immune-protection thresholds (70, 80, 90 and 100%).

Emirates	PPR Seroprevalence Pre-Vaccination (%)	PPR Seroprevalence Post-Vaccination (%)	Annual Increase (%)	Predicted Years to 70%	Predicted Years to 80%	Predicted Years to 90%	Predicted Years to 100%
Abu Dhabi	41.40	89.47	48.06	0.6	0.8	1.0	1.2
Dubai	46.67	96.67	50.00	0.5	0.7	0.9	1.1
Sharjah	55.17	98.26	43.09	0.3	0.6	0.8	1.0
Ajman	70.00	95.00	25.00	0.0	0.4	0.8	1.2
Umm Al-Quwain	50.00	100.00	50.00	0.4	0.6	0.8	1.0
Ras Al-Khaimah	77.07	96.00	18.93	0.0	0.2	0.7	1.2
Fujairah	64.12	97.65	33.53	0.2	0.5	0.8	1.1
All-UAE	52.32	93.77	41.45	0.4	0.7	0.9	1.2

## Data Availability

The original contributions presented in this study are included in the article. Further inquiries can be directed to the corresponding author.
